# Analysis of Multi-Target Synergistic Mechanism of Coix Seed Therapy for Herpes Zoster Based on Machine Learning and Network Pharmacology

**DOI:** 10.3390/genes16050580

**Published:** 2025-05-14

**Authors:** Zhiqin Song, Lin Yang, Jing He, Yuchao Li, Ningxian Yang, Min Yang, Mingkai Wu

**Affiliations:** 1 Institute of Crop Germplasm Resources/Institute of Modern Chinese Herbal Medicines, Guizhou Academy of Agricultural Sciences, Guiyang 550006, China; 2 Key Laboratory of Plant Resource Conservation and Germplasm Innovation in Mountainous Region, Ministry of Education, College of Life Sciences/Institute of Agro Bioengineering, Guizhou University, Guiyang 550025, China; 3 The Key Laboratory of Environmental Pollution Monitoring and Disease Control, Ministry of Education, Guizhou Provincial Engineering Research Center of Ecological Food Innovation, School of Public Health, Guizhou Medical University, Guiyang 561113, China; 4 The Rural Revitalization Service Center of Guiyang City, Guiyang 550004, China

**Keywords:** network pharmacology, machine learning, molecular dynamics simulation, herpes zoster, molecular docking

## Abstract

Objective: To explore the efficacy and mechanism of Coix seeds in treating herpes zoster (HZ) using an integrated computational approach. Methods: Network pharmacology, molecular docking, and machine learning were employed. Disease-related targets were collected from multiple databases, and intersection targets with Coix seed were analyzed via PPI, GO, and KEGG enrichment. A “TCM-Ingredient-Target” network was constructed using Cytoscape. Molecular docking and dynamics simulations were performed for validation. Results: Fifty-five overlapping targets were identified, with core targets including *TNF*, EGF, and *GAPDH*. Enrichment analysis revealed key pathways such as inflammation and immune regulation. Molecular docking confirmed strong binding affinity between active compounds and targets. Conclusions: This study demonstrates that Coix seed exerts anti-HZ effects through multi-target mechanisms, providing a theoretical basis for developing novel multi-pathway treatment strategies.

## 1. Introduction

According to the survey, the incidence of shingles in the world is 3–5% [1], with an annual increase of 2.5–5.0% in Asia and the Pacific [2,3]. Herpes zoster is caused by varicella-zoster virus (VZV), which has long been latent in the heel ganglia or cranial ganglia of the spinal cord and replicates in large numbers after activation when the body’s immunity is reduced [4]. In addition, VZV is also the main cause of primary varicella infection [5]. Its clinical manifestations include a painful local blister-like rash [6], and its complications, including post-herpes zoster pruritus, ulcerative keratitis, etc. [7,8], have a huge impact on patients’ mental and physical health. In recent years, from the simple relief of symptoms to early active intervention, studies have looked at the source to block the development of the disease. Within this trend, finding accurate and effective treatment targets for herpes zoster (HZ) and figuring out the mechanisms of occurrence and development of the disease have been the focus of medical research. This will teach us more about shingles and help develop effective new treatments.

Semen Coicis is the dry and mature seed of Semen Coicis, *Coix lacrma-jobi L. Viar. mayuen* (Roman.) Stapf. It is also known as the “green plant” [9], and is also known as six different meters, bodhi seed, fructus chinensis, Job’s tears, etc. [10]. In traditional Chinese medicine, Coix seed is considered to have a sweet taste, and be light and cold, with a variety of effects. The “Compendium of Materia Medica”, in Chinese medicine, records the following: It has the advantages of clearing heat and detoxification, reducing swelling and discharging pus, and promoting diuresis. It is often used for pain caused by dampness and aching muscles [11]. It can be used as a daily nourishing food because it is rich in various nutrients such as calcium, iron, phosphorus, B vitamins and amino acids necessary for the human body [12]. In modern medicine, Coix seed has multiple pharmacological effects, such as an anti-tumor effect [13], and an effect of lowering blood sugar and regulating lipid metabolism [14,15]. Among them, the effective components of Coix seed used in the treatment of cancer are Coix seed polysaccharide, triterpenes, starch and oil; in addition, these components also have anti-inflammatory, anti-allergic and anti-proliferation activities [16]. In terms of food, Coix seed is rich in various nutrients [17]. Coix seed is not only a traditional Chinese medicine but also a food [12]. It is increasingly widely used in the food industry [17]. It is commonly seen in Coix seed biscuits, Coix seed wine, Coix seed yogurt, Coix seed lactobacillus drink and other products [9,18,19]. This fully demonstrates that Coix seed not only has excellent food value, but also has a significant effect in medical care [15,20].

However, the specific mechanism of the active components of Coix seed on HZ has not been systematically clarified. Among them, machine learning is known for its powerful data processing capability [21,22], when dealing with large-scale and multi-dimensional data, showed unmatched advantages of traditional analytical methods, which can excavate potential key active ingredients from complex drug ingredients [23,24]. Through machine learning, network pharmacology and molecular docking technology, this study aims to reveal the potential mechanism of its multi-target, multi-pathway synergistic treatment of HZ, and provide a theoretical basis for the development of new natural drugs.

## 2. Machine Learning Screening Core Targets

### 2.1. Data Preprocessing and Model Training

In this study, we utilized machine learning techniques to analyze shingle-related data sourced from the GEO database. Our primary objective was to pinpoint key genes that play a role in the interaction between Coix seed components and herpes zoster. In addition, we aimed to assess the effectiveness of various algorithms in identifying these genes. The experimental data obtained from GEO data library (https://www.ncbi.nlm.nih.gov/geo/, accessed on 1 March 2025), including the design documents and gene expression matrix list, are stored in TAB delimited text format.

### 2.2. Data Reading and Integration

The read.table function is used to read the expression matrix, design information, and gene list. By screening the genes in the reserved gene list and transposing the expression matrix, the samples were treated as rows and genes as columns, with a focus on genes potentially interacting with Coix seed components in the context of herpes zoster.

### 2.3. Sample Type Annotation

The sample type information from the design file is integrated into the representation matrix to generate a new data box containing the sample type.

### 2.4. Data Set Partitioning

The createDataPartition function was used to divide the data set into a training set (70%) and a test set (30%) to ensure the independence of model training and evaluation. This division ensured the independence of model training and evaluation, which is crucial for developing models that accurately predict the interaction between Coix seed components and herpes zoster-related genes.

### 2.5. Model Training

The caret package was used to train eight machine learning models, including random forest (RF), support vector machine (SVM), Generalized linear Model (GLM), gradient elevator (GBM), K-nearest neighbor (KNN), neural network (NNET), LASSO regression (LASSO), and decision tree (DT). By comparing these models, we can select the optimal one for identifying genes that mediate the interaction between Coix seed components and herpes zoster, thereby gaining insights into potential therapeutic targets.

## 3. Network Pharmacological Analysis

### 3.1. Target Screening

The TCMSP database (https://ngdc.cncb.ac.cn/databasecommons/database/id/4096, accessed on 2 March 2025) was used to obtain nine accords with a condition, and the chemical composition of semen coicis is obtained from the literature. Then, the SMILES number of the Coix seed ingredient was obtained through the organic small molecule Bioactivity database (PubChem), and the structural formula of TCM active ingredient was imported using the SwissTarget Prediction platform. The species was homo sapiens. The screening condition is “Probability > 0” of the active ingredient acting on the target, and the target of the potential active ingredient can be predicted.

### 3.2. Acquisition of Disease Target and Intersection Target

At DrugBank (https://go.drugbank.com/, accessed on 9 March 2025), GeneCards (https://www.genecards.org/, accessed on 9 March 2025), OMIM (https://www.omim.org/, accessed on 9 March 2025), PGKB (https://www.pharmgkb.org, accessed on 9 March 2025) In the four databases, the keyword “Osteoarthritis” was used to search, and the obtained genes were standardized in the Uniprot database, and the herpes zoster target library was established after de-duplexing. Through the microscopic letter online platform (https://www.bioinformatics.com.cn/, accessed on 9 March 2025), potential semen coicis targets for screening, herpes zoster take overlaps between related gene targets, effective ingredients of semen coicis–zoster intersection targets, and the Wayne map were found

### 3.3. Construction of “Chinese Medicine Ingredients-Target” Network

Cytoscape 3.8.0 software was used to import the prepared compound gene file, conduct network topology analysis, and adjust the target graph, color, transparency, and size in reference to the Degree value, so as to construct the “Chinese medicine Composition-Target” network diagram.

### 3.4. Protein Interaction (PPI) Network Construction and Topological Analysis

The String platform (https://string-db.org/, accessed on 9 March 2025) was used to import common genes, screen the results with the highest confidence of 0.400, and exclude the results of free gene nodes; that is, the protein interaction relationship. We imported the result into Cytoscape 3.8.0 software and selected the “network analyzer” tool to obtain network topology parameters, and drew the PPI graph.

### 3.5. Functional Enrichment Analysis of Gene Ontology (GO) and Pathway Enrichment Analysis of Kyoto Encyclopedia of Genes and Genomes (KEGG)

Using the DAVID (https://david.ncifcrf.gov/, accessed on 9 March 2025) platform for GO and KEGG enrichment analysis of intersection targets, and the data import microscopic letter online platform to realize data visualization, the drawing of a histogram, the bubble chart, and analysis of Coix seed treatment of herpes zoster targets of biological function, we explored the potential biological pathways and signaling pathways.

### 3.6. Molecular Docking Verification

The four core active ingredients with the highest degree value were selected as small molecule ligands, and the six core targets with the largest degree value were selected as protein receptors, and pairwise docking verification was conducted between ligands and receptors. In order to make the protein receptor structure more accurate, the core target gene name was first searched in Uni prot database, human was selected, and the protein ID serial number was obtained. The ID was entered into the RCSB PDB (https://www.rcsb.org/, accessed on 12 March 2025) database for retrieval, and the 3D protein structure was obtained. From the Pub Chem (https://pubchem.ncbi.nlm.nih.gov/, accessed on 12 March 2025) database, key components of semen coicis small molecule ligands 2 d structure were obtained. The obtained receptor pdb format files and ligand mol2 format files were imported into the Schrodinger 2021 software, the protein receptor dewater and deligand processing were performed, the active pocket was determined, the pdbqt files for the ligand and receptor were exported, and the docking results were imported into PyMOL (PyMOL 2.5.0) software for visualization processing, such as amino acid residues showing hydrogen bonds and their functions.

### 3.7. Molecular Dynamics Simulation

The active ingredient and target with the lowest binding energy in the molecular docking results were selected for molecular dynamics simulation, to explore the binding situation of protein and small molecules, and to evaluate the interaction between them. The system used in this study is a protein–small molecule complex consisting of a target protein molecule and an active component small molecule [25], which was simulated using Gromacs (Gromacs 2024.3) software [26].

## 4. Result

### 4.1. Machine Learning Method

**Figure 1:**
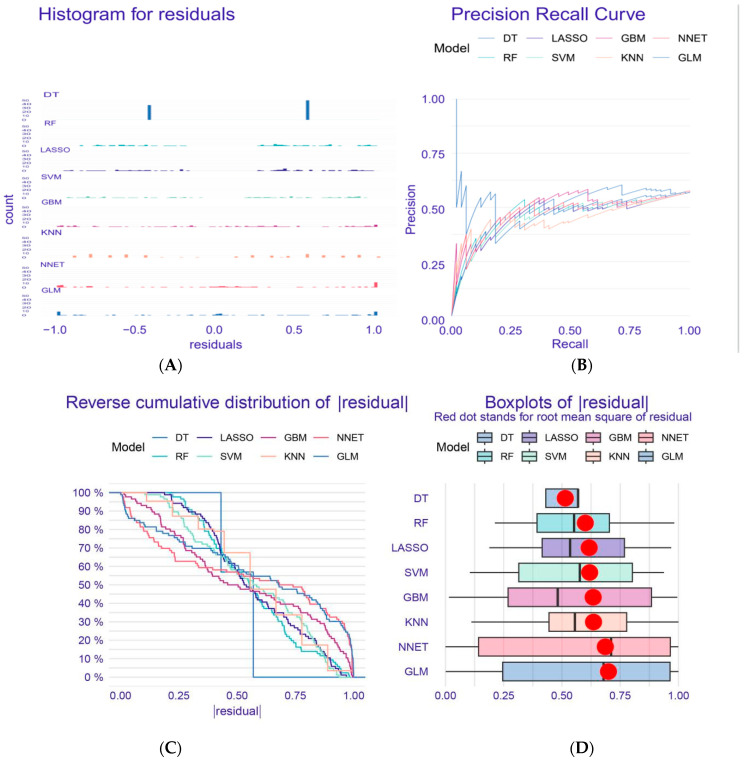

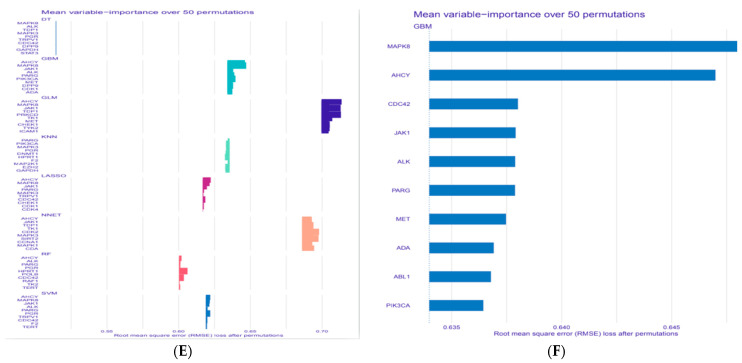
Machine learning model diagram. (**A**). Residual Histogram: displays the distribution of residuals for the model; (**B**). Precision-Recall Curve: illustrates the trade-off between precision and recall for different models; (**C**). Reverse Cumulative Distribution of Residuals: shows the reverse cumulative distribution of residuals, with a red dot indicating the root mean square of residuals; (**D**). Residual Boxplot: presents the boxplot of residuals for various models; (**E**). Variable Importance Across Models: displays the relative importance of variables for all models. (**F**). DT Model Variable Importance: shows the importance of variables specifically for the DT model.

In this study, we applied a variety of machine learning methods to select feature genes. First, Figure 1C shows the inverse cumulative distribution of absolute residual values. It can be seen from the figure that the faster the curve of the model declines, the smaller the absolute values of most residual errors in the model become, and the closer the predicted values of the model become to the real values. The curve of the DT model drops relatively fast, which indicates that the residual control is better. Figure 1D shows the distribution of the residuals of each model. The residuals of the DT model are relatively concentrated near 0, so the prediction effect may be better. It can be seen from the precision recall curve that the DT model has higher precision in the early stage. Figure 1E includes a variety of models. Different models have different combinations and importance degrees for variables with greater influence, indicating that different models have different sensitivity and dependence degrees on variables. It can be seen from the figure that the DT model is the most suitable model. Figure 1F shows the GBM model. It can be seen from the figure that the bar shape corresponding to *MAPK8* and *AHCY* is significantly longer than other variables, indicating that these two variables have the greatest impact on the model prediction results and are the most critical variables in the GBM model. *PIK3CA* corresponds to the shortest bar, indicating that it is the least important to the model.

### 4.2. The Component Target of Coix Seed

Nine active ingredients with OB > 30% and DL > 0.18 were obtained from the TCMSP database. The chemical composition of Coix seed was obtained by searching the literature. A SMILES number could not be obtained through pubchem for some of the chemical components, and the target of the remaining components could be predicted by SwissTarget Prediction after obtaining SMILES numbers through pubchem. The remaining 19 components had 503 targets.

### 4.3. Drug Component Targets

**Figure 2:**
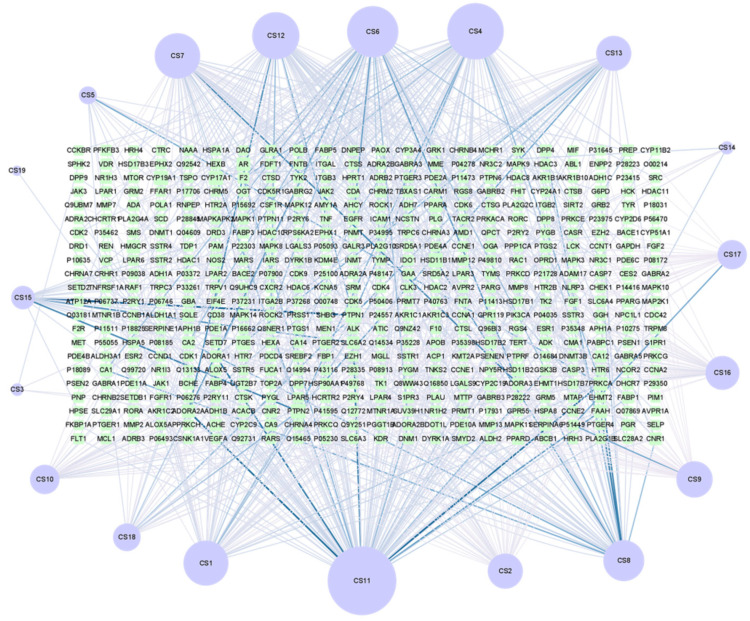
Drug ingredients of Coix seed—target diagram, in which the circle is the drug ingredients, the square target name, and the line segment represent the interrelation between them.

The Active ingredient-target map was constructed using Cytoscape 3.8.0 with 503 nodes and 822 edges (Figure 2). The larger the node, the more important it is. The more lines, the closer the connection. The top active ingredients are glycerol monooleate, squalene, glycerol monooleate, and 6′-O-ferulic sucrose.

### 4.4. The Target of Disease Action and Intersection Target Were Obtained

**Figure 3:**
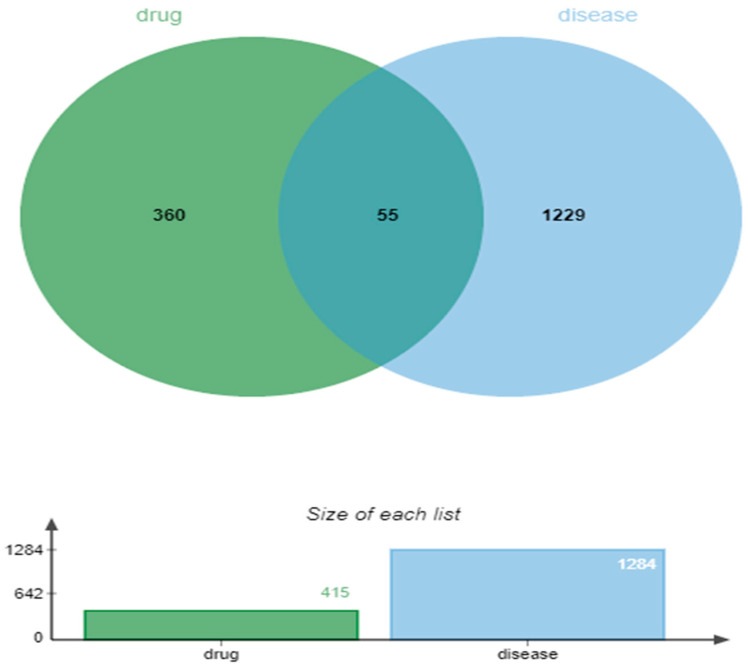
Venn diagram; green represents drug targets and blue represents disease targets.

A total of 832 disease targets were collected from the GeneCards database, 288 from the OMIM database, and, finally, 7 targets were collected from the DrugBank database. By combining the results of the above three databases and eliminating duplicate genes, a total of 1284 herpes zoster targets were obtained. Using Weisheng online cloud platform, 55 intersection targets were obtained (Figure 3).

### 4.5. PPI Network and Key Target Analysis

**Figure 4:**
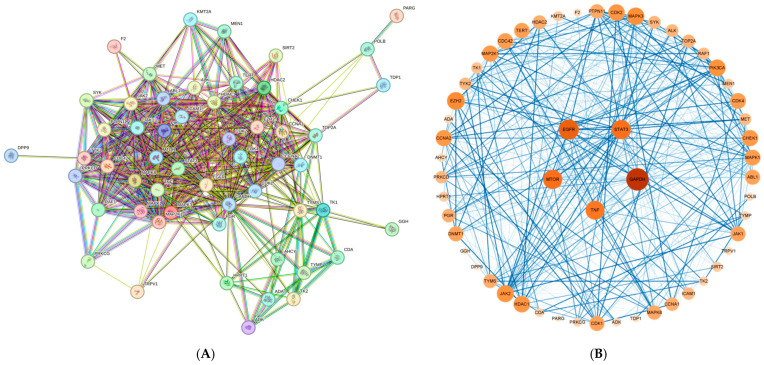
(**A**) is the protein interaction map obtained from the String database, and (**B**) is the protein interaction map obtained by Cytoscape 3.8.0 modification.

The intersection targets of Coix seed components and diseases were imported into the String database, and the species was set to human with a confidence of 0.4 to obtain the interaction between proteins. In addition, 55 nodes and 494 edges were obtained in Cytoscape 3.8.0. The five targets with the highest degree value were *GAPDH*, *TNF*, *EGFR*, *STAT3* and *MTOR* (Figure 4).

### 4.6. GO Enrichment Analysis

**Figure 5:**
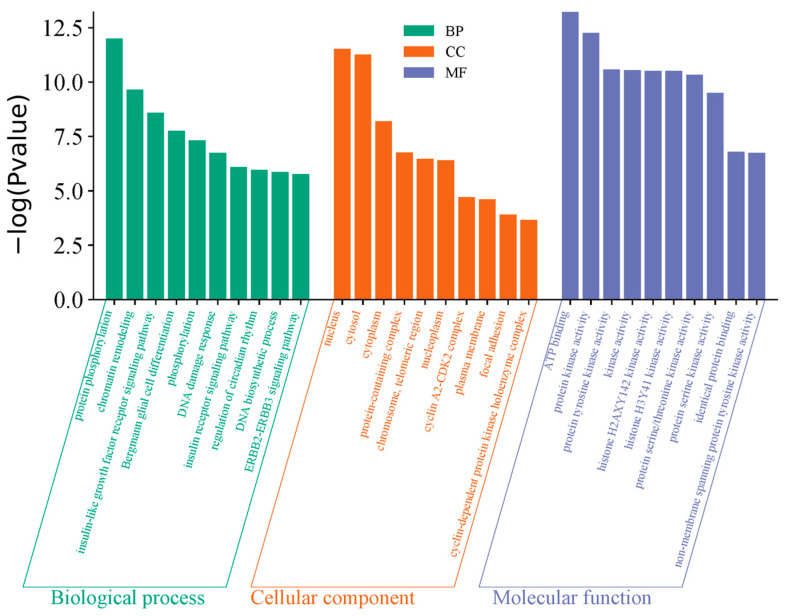
GO enrichment analysis.

The biological process (BP), cellular component (CC), and molecular function (MF) of Coix seed were enriched and analyzed with the DAVID database for 55 intersection targets of HZ. A total of 290 BP items, 51 CC items and 109 MF items were obtained, and the *p*-values corrected by −log^10^ function were screened from high to low. Finally, the top 10 results of BP, CC, and MF were selected, respectively, and the results were visualized by drawing on Weichenxin platform (Figure 5).

### 4.7. KEGG Enrichment Analysis

**Figure 6:**
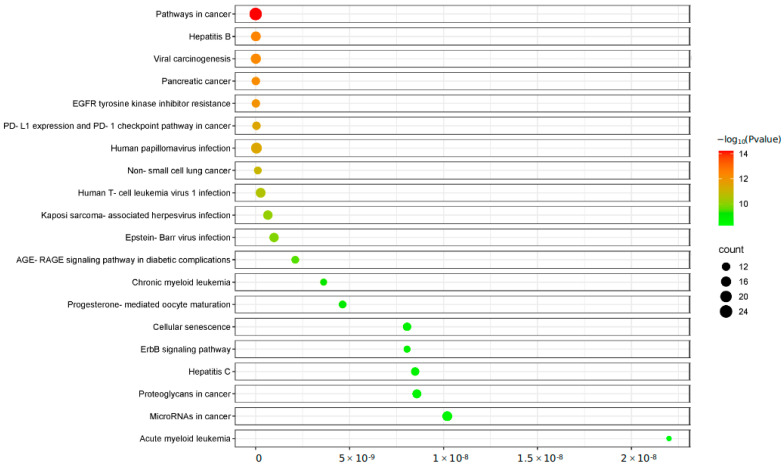
KEGG enrichment pathway.

Similarly, KEGG signaling pathways were analyzed for targets related to shingles in Coix seed through DAVID database, and a total of 21 participating signaling pathways were obtained (Figure 6). According to the corrected *p*-value of the −log^10^ function, a previous significant path is screened. These pathways are mainly involved in cancer-related pathways, apoptosis, endocrine resistance and so on.

### 4.8. Pathway–Target–Active Ingredient Diagram

**Figure 7:**
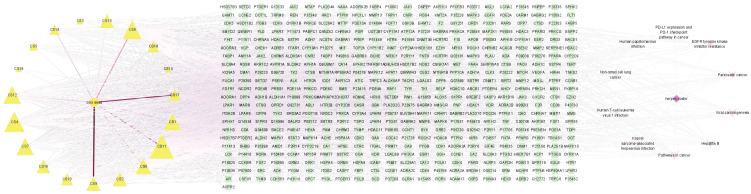
Active ingredient diagram of the pathway target, in which an equilateral triangle represents the signaling pathway and disease, a rectangle represents the target, and an inverted triangle represents Chinese medicine and its corresponding active ingredient.

The top 10 pathways and targets in the enrichment results of KEGG pathway were selected and imported into Cytoscape3.8.0 software to plot the “TCM-component-target-pathway” network diagram (Figure 7). A network diagram with 517 nodes and 975 kinds of interaction associations was obtained, in which the equilateral triangle represents signal pathway and disease, and the rectangle represents target. The inverted triangle represents Chinese medicine and corresponding active ingredients based on analysis of the top 10 signaling pathways and the top 19 drug components with degree values, respectively. The pathways were pathways in cancer, Hepatitis B, viral carcinogenesis, pancreatic cancer, and *EGFR* tyrosine kinase inhibitor resistance, PD-L1 expression and PD-1 checkpoint pathway in cancer, human papillomavirus infection, non-small cell lung the active components of cancer, human T-cell leukemia virus 1 infection, Kaposi sarcoma-associated herpesvirus infection are shown in Table 1. It is suggested that the above signaling pathways and effective components may play a greater role in the treatment of herpes zoster with the effective components of Coix seed.

### 4.9. Molecular Docking Verification Results

**Figure 8:**
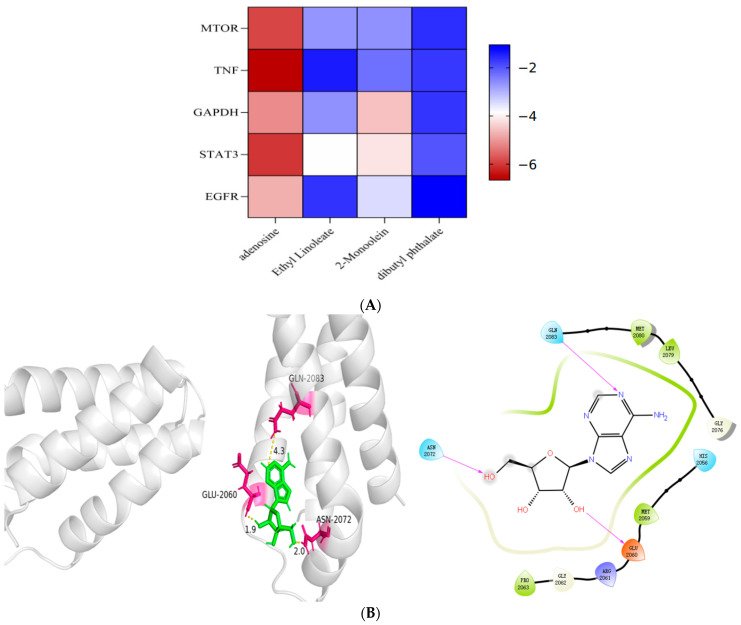

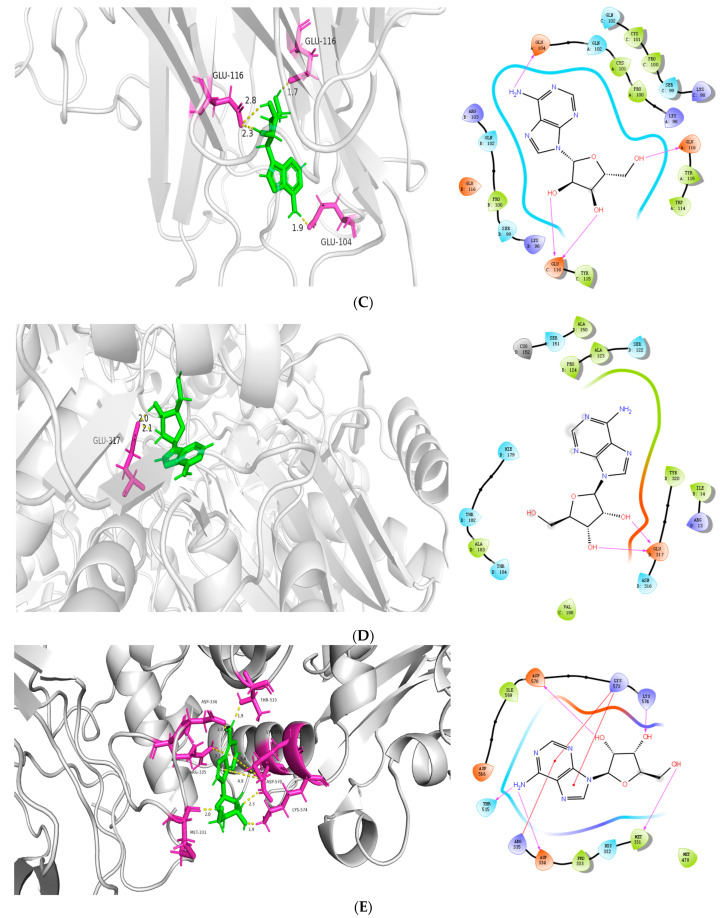

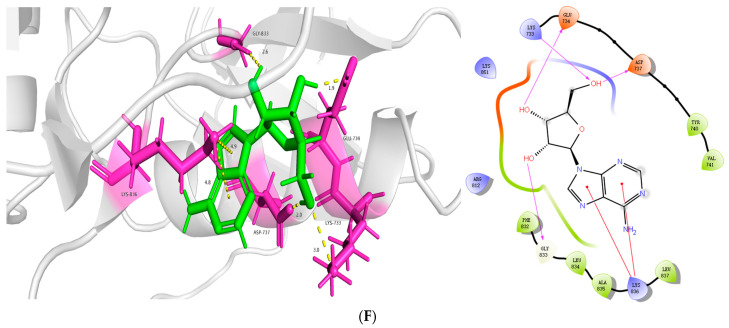
(**A**): Molecular docking score heat map showing the binding affinity of each target to the ligand. (**B**–**F**) are the molecular docking 2D interaction diagram and visual display of *MTOR*, *TNF*, *GAPDH*, SPT3, and ZGFRand adenosine, respectively.

Molecular docking is mainly used for structural docking of small molecules and target proteins, and to evaluate their affinity to specific binding sites. The receptor selected five core targets, and the ligand selected the top four active ingredients. If the binding energy is less than 0, it means that it can be connected under natural conditions, and if it is less than −5 kJ/mol, it means that the docking result is good. The two-dimensional diagram with good docking results is visualized (Figure 8).

### 4.10. Molecular Dynamics Simulation

**Figure 9:**
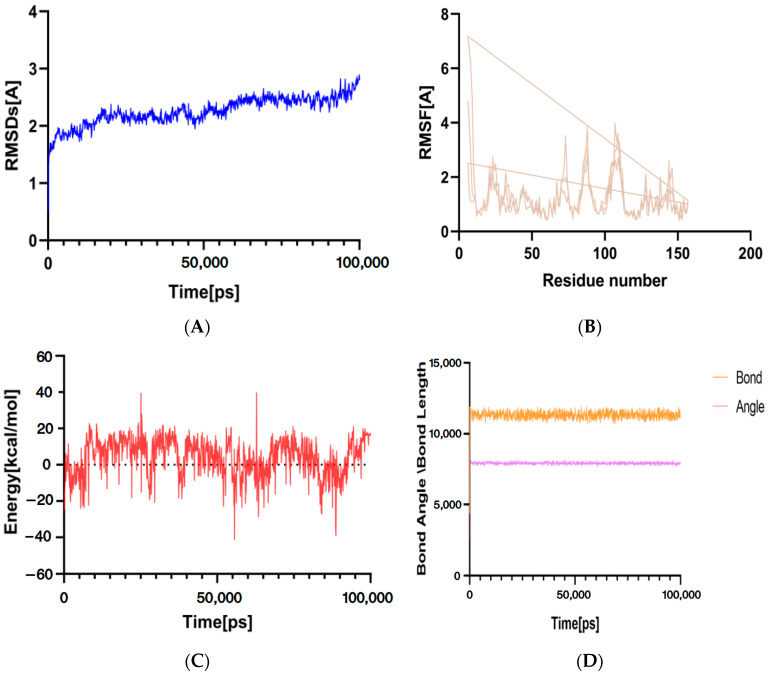
(**A**): RMSD change curve; (**B**): fluctuation of amino acid residues; (**C**): changes in ability; (**D**): atomic and bond length curves.

Molecular dynamics techniques were used to simulate the stability and flexibility of Coix seed adenosine and herpes zoster *TNF* genes to evaluate their interaction. The RMSD value reflects the fluctuation degree of protein conformation and can be used to evaluate the stability of ligand binding to target protein. The smaller the value is, the more stable the binding is. As shown in Figure 9A, the RMSD value of the complex fluctuates little, which proves that the complex is in a relatively stable state during the simulation. RMSF reflects the fluctuation of protein amino acid residues, and most residues have higher RMSF value, indicating higher flexibility (Figure 9B). Ability change is a key part of understanding the behavior and interaction of molecular systems. As shown in Figure 9C, although there are ups and downs, there is no continuous upward or downward trend, indicating that the simulation system is relatively stable and the simulation process is reasonable. It can be seen from Figure 9D that both the bond length curve (orange) and the bond angle curve (purple) show relatively stable fluctuations during the simulation time, and there is no large abrupt change or continuous upward or downward trend. This shows that the molecular bond length and bond angle are stable in the simulation system, the molecular structure does not change dramatically, and the simulation process is reliable.

## 5. Discussion

Shingles is a common viral infection characterized by a painful, localized, blistering rash [5]. At present, the treatment of HZ involves reducing symptoms and shortening the duration of onset by using antiviral drugs during acute onset, but these do not reduce the risk of onset and complications of HZ [27]. This highlights the urgent need for novel multi-target therapies derived from natural products. Our study systematically elucidated the multi-component and multi-target mechanism of Coix seed’s therapeutic effect. Based on network pharmacology, machine learning and molecular docking analysis, the active components of Coix seed were studied.

Machine learning is a powerful tool for data analysis and is gradually showing its interdisciplinary advantages in the field of drug research [28]. This method is very innovative and has great potential in practical applications. In this study, a variety of machine learning algorithms are used to carry out feature selection, which greatly improves the reliability and accuracy of the results. Different from traditional statistical methods, machine learning algorithms have significant advantages in processing complex biological data [29]. In addition, the integration of multiple algorithms can effectively avoid the overfitting risk caused by using a single algorithm [30].

Network pharmacology identified 55 HZ-related targets, with *GAPDH*, *TNF*, *EGFR*, *STAT3*, and *MTOR* as core mediators. *GAPDH* acts as a cellular metabolic protein [31]. Dilawari et al. [32] explored the possibility of *GAPDH* as a potential target for antiviral therapy, and expounded the functions and related mechanisms of *GAPDH* in the course of multiple viral infections. It provides a theoretical basis and research prospect for studying the relationship between herpes zoster virus and *GAPDH*. *TNF* is a highly pro-inflammatory cytokine that not only helps regulate the immune response, but also contributes to the development of severe inflammatory diseases [33]. Internal rheumatoid arthritis has been found to have a higher risk of HZ [34]. Studies on human lung embryonic fibroblasts (HEL) have shown that *TNF*-α activation completely blocks antigenic expression and replication of VZV [35]. Therefore, patients treated with *TNF* inhibitors may develop varicella infection or latent VZV activation [36]. Zhang et al. [37] indirectly elaborated the relationship between shingles and *EGFR*, suggesting a relationship between *EGFR* and related treatment and skin symptoms in skin diseases, which has a certain reference value for studying the potential relationship between shingles and *EGFR*. Sen et al. [38] found that VZV could phosphorylate *STAT3* in infected cells in vitro and human skin xenografts of SCID mice, thereby inducing anti-apoptotic protein survivin. Thus, the control of this cell signaling pathway is critical to the pathogenesis of herpesvirus, and *STAT3* activation and survivin upregulation are important common mechanisms [38].

GO enrichment analysis revealed that the active components of Coix seed are involved in cell reaction, proliferation and chromatin remodeling, which are essential for the viral infection of VZV. Molecular functions such as protein kinase activity and enzyme binding are further involved in intracellular signal regulation, which is consistent with the antiviral and immunomodulatory effects of Coix seed. KEGG enrichment analysis showed that although our study provided mechanistic insights into the multi-target action of the active components of Coix seed, limitations still exist. Due to the lack of in vivo validation, future experimental confirmation of target involvement and pathway regulation is needed. In addition, the pharmacokinetic interaction of Coix seed has not yet been explored. However, these findings lay the foundation for the development of therapeutics based on the active ingredients of Coix seed that address both the antiviral and immunomodulatory effects of VZV, a paradigm shift from a single-target approach. Future work should delineate the temporal regulation of these pathways at various stages of VZV.

The relationship between the core active ingredient and the key target was verified by molecular docking, and the results showed that the binding stability between the ligand and the receptor was good. The molecular dynamics simulation of the effective component of Coix seed with high score-HZ was carried out, which was consistent with the docking results. RMSD and RMSF analysis revealed the structural dynamics and stability of the complex. Both the bond length and the bond angle show relatively stable fluctuation state, which indicates that the molecular structure does not change dramatically, and the simulation process is reliable.

## 6. Conclusions

Through network pharmacology, machine learning, molecular dynamics simulation and molecular docking, this study systematically revealed the potential therapeutic mechanism of the active ingredients of Coix seed for herpes zoster. Machine learning and network pharmacological analysis showed that Coix acts on a wide range of targets, involving multiple HZ-related biological pathways, especially cancer pathways. Molecular docking verification further confirmed the strong binding force between the key active ingredients and the HZ core target. Molecular dynamics simulations are all about evaluating the interaction between the two. The stability and flexibility of Coix seed adenosine and herpes zoster *TNF* gene were obtained. These results not only provide theoretical support for the potential of Coix seed in HZ therapy, but also lay a foundation for its multi-target and multi-pathway treatment strategy. In addition, the combination of network pharmacology and machine learning technology provides a new prospect and method for TCM prevention and treatment of diseases. However, this study has limitations: (1) The predictions require experimental validation (e.g., in vitro/vivo assays) to confirm target modulation and pathway regulation; (2) The pharmacokinetic properties (e.g., bioavailability, metabolism) of Coix seed components were not addressed, which are critical for translational applications. Despite these constraints, our findings provide a theoretical foundation for developing multi-target HZ therapies from Coix seed and exemplify the utility of computational methods in traditional medicine research.

## Figures and Tables

**Table 1:** Code name mapping table.

Code	Corresponding Name
CS1	(2r)-2,3-Dihydroxypropyl (9z)-Octadec-9-Enoate
CS2	(3R,8S,9S,10R,13R,14S,17R)-17-[(2R,5R)-5-ethyl-6-methylheptan-2-yl]-10,13-dimethyl-2,3,4,7,8,9,11,12,14,15,16,17-dodecahydro-1H-cyclopenta[a]phenanthren-3-ol
CS3	(6Z,10E,14E,18E)-2,6,10,15,19,23-hexamethyltetracosa-2,6,10,14,18,22-hexaene
CS4	2-Monoolein
CS5	6′-O-feruloylsucrose
CS6	adenosine
CS7	alpha1-Sitosterol
CS8	caprolactam
CS9	cholesterol
CS10	coixenolide
CS11	dibutyl phthalate
CS12	ethyl Linoleate
CS13	inosine
CS14	rossicasin A
CS15	sibiricose A3
CS16	stigmasterol
CS17	sucrose
CS18	uridine
CS19	zarzissine

## Data Availability

The original contributions presented in this study are included in the article. Further inquiries can be directed to the corresponding authors.
